# A study on the evolution of economic patterns and urban network system in Guangdong-Hong Kong-Macao greater bay area

**DOI:** 10.3389/fpubh.2022.973843

**Published:** 2022-10-25

**Authors:** Bo Tang, Zehui Chen, Yuanyuan Zhang, Hua Sun

**Affiliations:** ^1^School of Resources and Planning, Guangzhou Xinhua University, Guangzhou, China; ^2^Faculty of Innovation and Design, City University of Macau, Macau, China

**Keywords:** economic pattern, urban network, gravity center model, social network, Guangdong-Hong Kong-Macao greater bay area (GBA)

## Abstract

The COVID-19 pandemic has seriously affected China's macroeconomy, industrial transformation, and high-quality development. Research on economic patterns and urban network systems can provide a reference for healthy development of the regional economic system. The evolution of the economic pattern and urban network system of Guangdong-Hong Kong-Macao Greater Bay Area (GBA) from 2010 to 2020 is investigated using methods (e.g., the gravity center model, the gravitational force model, social network analysis, and geographic information system). (1) The gravity center of gross domestic product (GDP) of the GBA is located in Nansha district, Guangzhou, with a skewing direction northwest-east-northwest and a movement rate of “large-small-large.” The center of import and export and the center of consumption show a “zigzagging migration” in which the center of investment shows an “irregular (random) migration”. (2) The economic connection degree of cities in the GBA exhibits a high ascending velocity, and the whole area tends to be mature, with a significant effect of spatial proximity. With the steady increase in network density, there is significant polarization of network centrality in the region. The four major cohesive subgroups have been relatively stable and consistent with the degree of geographic proximity of the cities. The center-periphery structure is more significant, in which the core area is extended to the cities on the east coast of the Pearl River Estuary, thus forming the core cluster of “Hong Kong-Shenzhen-Guangzhou-Dongguan.” In this study, the evolution of economic patterns and urban network systems in the GBA over the past decade is analyzed using multiple methods (i.e., gravity model, urban network system analysis, and geographic information system) based on urban socioeconomic data by starting from various spatial elements (e.g., “points, lines, and networks”) to gain insights into and optimize research on regional economic development after the COVID-19 pandemic.

## Introduction

At present, China's economy is shifting from high-speed growth to high-quality development, which has become the fundamental goal and core requirement of building the country with Chinese characteristics in the new era (1). The smooth functioning of the economy and the harmonious development of cities are critical prerequisites for China's high-quality development. In the context of economic globalization, different regions (cities) are having an increasingly close economic connection such that the optimization and adjustment in the regional spatial structure are of increasing importance (2). Regional economic pattern is a relative locational relationship and a distribution form of regional economic elements. It has been the most intuitive expression of human economic activities and locational choice, and it has been the key content in the research conducted on regional economics and economic geography (3, 4). From the early 19th century to the 1940 s, regional economic space was established primarily based on locational choices, spatial behavior, and organizational structure of industries and enterprises in the microscale research stage (5). In the 1980 s, after World War II, the research on the overall spatial structure and evolution of the region largely focused on the mesoscopic scale (6, 7). After the 1980 s, in the stage of new spatial economics with the orientation of economic globalization and unique economic geography, the respective stage had the significant characteristics of time, research theme, and research focus (8, 9). It changes from the initial abstract theoretical research to empirical research that seeks the optimal spatial combination and differentiation of economic agents. Classical location theory, modern location theory, regional spatial structure and its evolution theories (e.g., growth pole theory, core-edge theory, point-axis theory, circle theory, and network structure theory) (10), new economic geography (11), and other classical theories and research paradigms have been established (12, 13). Trends regarding the clustering and spreading of economic activities in the regional space affect the regional economic growth and changes in the development gap, and adjustments and changes are constantly available in the regional economic and spatial structures (14, 15). Thus, numerous achievements and empirical cases regarding the regional economic and spatial structure have been achieved [e.g., the characteristics revealed by the spatial correlation about the evolution of regional patterns (16, 17), as well as the framework and role mechanism of the multifaceted regional economic space (18)]. The focus are placed on the interactive relationship between urban spatial structure and coordination and development of the regional economy (19, 20). The intensity of economic connection (21) and the trend expressed by the evolution of urban network systems are stressed (22). Moreover, the urgency and uncertainty characterizing major public emergencies significantly hinder the development of the global economy. The effects of public health events on macroeconomics, financial risk regulation, trade markets, and economic governance have been investigated extensively (23, 24).

The economic gravity center is a vital manifestation of the pattern of regional economics, and scholars have strived to investigate regional economic and social issues in accordance with the theory of the economic gravity center. For instance, Zhou conducted an empirical study on the relationship among the economic center in China, regional disparity, and harmonious development. He highlighted that China's economic center generally moves southward, and that the regional disparity between the north and the south extends increasingly (25). Over the past few years, population centers (26), food production centers (27), transportation centers (28), environmental pollution centers (29), tourism centers (30), and others have been studied such that the spatial pattern of the regional economy has been investigated from different perspectives and on different scales. Moreover, along with the development of regional economic integration and specialization of industrial division, the economic linkages between cities will be closed, and the flow of factors will be smoother, which is organized by the regional spatial organization with “in spatial flow” as the logic. Some changes may occur in the development pattern of urban networking, thus leading to change in the depth and breadth of regional socioeconomic spatial structure (31, 32). The connotation characteristics, formation mechanism, and development trend of urban network systems have been explored. As a result, the above achievements provide a reference and lay a solid basis for further studies. Moreover, studies on the spatial structure of regional economics should be urgently developed from single-factor to multifactor analysis gradually, stress the multi-scale extension, strengthen the dynamic process, deepen the evolution analysis (33, 34), and emphasize the integration with the methods of spatial measurement, spatial analysis, social network, etc. (35).

Urban agglomerations have become the major spatial form of urbanization, and the development of cities and urban agglomerations will be the direct driving force of changes in regional economic patterns. The city, the political, economic, and cultural center of the region, has become more and more closely correlated with other cities in the vicinity of the region, and several cities of different sizes and functions together form an urban system with a certain spatial structure. The respective city is a member of the whole, and together they maintain the coordination and stability of the urban system and promote the rapid development of the regional economy. When the external environment of the urban system changes (e.g., world economic crisis and public health events), the internal conditions change, thus leading to a change in the way of economic activity of the city. The old equilibrium within the urban system begins to break down, and the structure of the urban system evolves. The spatial structure of the GBA is analyzed through the economic center of gravity and urban network, and the spatial structure of the “core-periphery” urban network and the coexistence of networked and centralized features are explored. Moreover, for various cities, the improvement of regional network status can enhance their strength and connection with core cities, thus promoting the healthy and coordinated development of the regional urban system. Moreover, against the background of drastic changes in the global economic environment and the normalization of COVID-19, the exploration of changes in the economic pattern and urban network system of the GBA has strategic significance in the formulation of policies that are consistent with future trends and is capable of facilitating harmonious regional and urban development. Accordingly, the changing trends of the economic center, import and export center, consumption center, and investment center from 2010 to 2020 are analyzed in this study using the gravity model, gravitational model, social network analysis, and GIS. Moreover, the changing trends of the urban network system are analyzed from the perspective of economic connection, which can be used to provide a reference for the harmonious and co-integrated regional development of GBA after the COVID-19 pandemic.

## Methods

**Figure 1:**
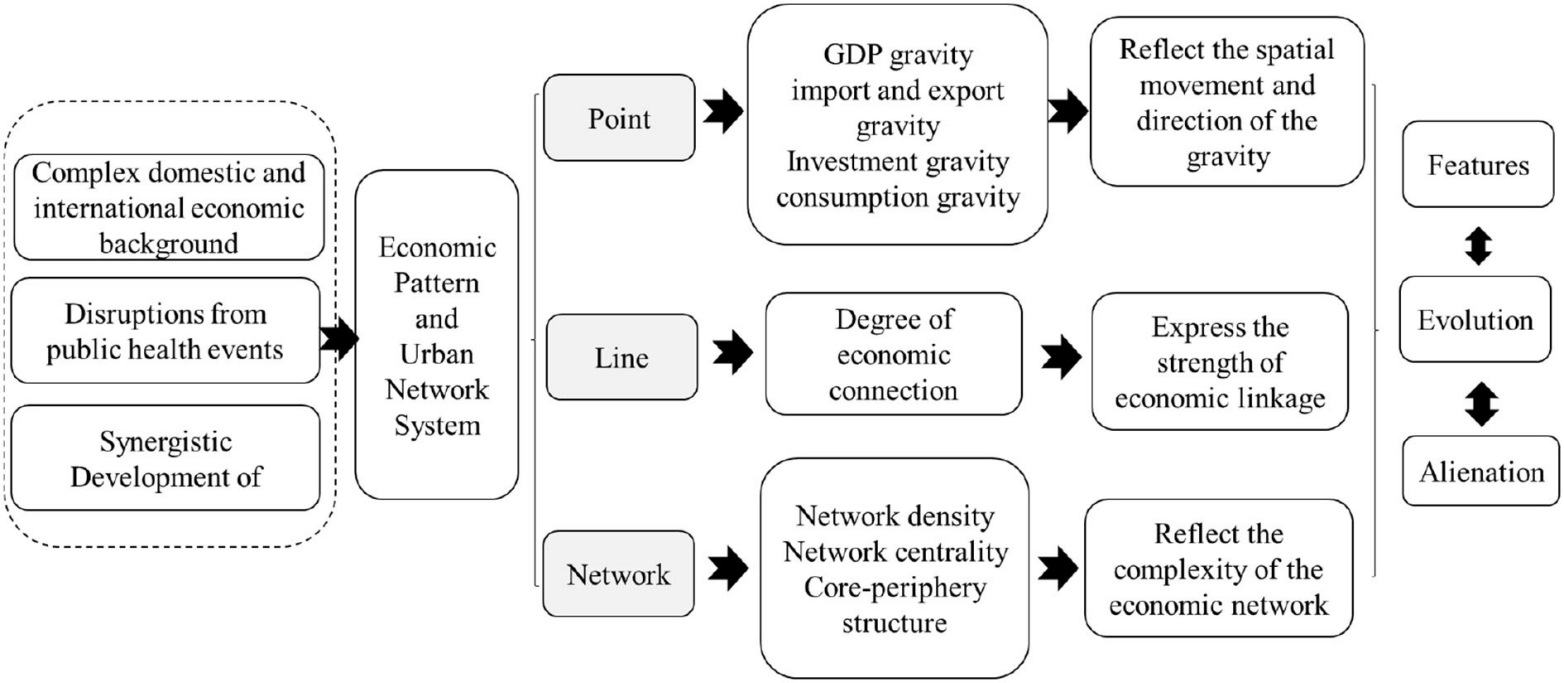
Methodological framework.

The regional economic structure is the spatial layout and interrelationship of economic activities in the region, and the economic spatial agglomeration and diffusion between economic units in the form of different factor flow, which is a comprehensive spatial pattern of the regional central city and peripheral circle cities; it is the correlation among points, lines, surfaces, and networks (36). This study focuses on the spatiality of the regional economic structure from the perspective of geography and economics and tries to sort out the characteristics, evolution, and alienation of the regional economic spatial structure of the GBA in the past 10 years from the elements of “point-line network” and establish the methodological framework of this study, as presented in Figure 1. The existing research and literature have suggested that the points primarily indicate the spatial movement and directionality of the center of gravity of GDP, import and export, investment, and consumption in the GBA from the perspective of the gravity center of economy (37). The lines and networks investigate the spatial complexity and interaction from four aspects: economic connectivity, network density, network centrality, and core-periphery (38, 39).

### Gravity model

The center of gravity calculation model was initially proposed in 1874 (40). Assuming the scale-free property, the gravity model is a widely used approach for estimating and predicting urban mobility networks at certain levels of aggregation (41). The model has been extensively studied in the problem of balanced economic development and regional economic spatial evolution trajectory (42). However, the characteristics of economic patterns and urban networks might vary depending on the spatial and temporal resolutions of data (e.g., population flow, traffic flow, capital flow, and information flow). In this study, from a macro-middle scale and focusing on the level of economic development and factors, the center of gravity coordinates, moving distance, and moving direction of the economic pattern of GBA are reflected from four perspectives: economic center of gravity, import and export center of gravity, investment center of gravity, and consumption center of gravity (37).

#### Gravity coordinates

The gravity center is the equilibrium point of economic moments in the study region. Assuming that there are *n* cities in the area, the center coordinates of the *i* city are *X*_*i*_ and *Y*_*i*_, and *P*_*i*_is the value of some attributes of the *i* city (e.g., economy, import/export, and consumption). The geographical coordinates of the gravity center are expressed as follows:


(1) $$\begin{array}{l} x^{*}=\overset{n}{\underset{i=1}{\sum }}P_{i}x_{i}/\overset{n}{\underset{i=1}{\sum }}P_{i}\;y^{*}=\overset{n}{\underset{i=1}{\sum }}P_{i}y_{i}/\overset{n}{\underset{i=1}{\sum }}P_{i} \end{array}$$

#### Gravity shift

The shift of the gravity center is the degree of deviation of the gravity center in two different periods and determines whether a particular indicator is unbalanced in the region. It is assumed that *a* and *b* denote additional years such that the measure of the distance of gravity center shift is defined as:


(2) $$\begin{array}{l} x^{*}=\overset{n}{\underset{i=1}{\sum }}P_{i}x_{i}/\overset{n}{\underset{i=1}{\sum }}P_{i}\;y^{*}=\overset{n}{\underset{i=1}{\sum }}P_{i}y_{i}/\overset{n}{\underset{i=1}{\sum }}P_{i}\\ D_{a-b}=R\sqrt{{\left({x_{a}}^{*}-{x_{b}}^{*}\right)}^{2}+{\left({y_{a}}^{*}-{y_{b}}^{*}\right)}^{2}} \end{array}$$

#### Gravity direction

The direction of the gravity center is expressed for consistency change, usually expressed as the angle θ between the two centers of gravity in comparison with the previous time unit displacement. The larger the θ, the more significant the change difference will be. Moreover, since θε[0,180°], it employs its cosine value to express the change consistency index *C*. The more considerable of *C*, the greater the change will be. If the change in the latitude and longitude of the gravity center is Δ_*x*_ and Δ_*y*_in the last time unit, the consistency of the change in the gravity center of the economy can be expressed as:


(3) $$\begin{array}{l} C=\cos\theta \\ =\left[\left(\Delta x_{1}\Delta x_{2}\right)+\left(\Delta y_{1}\Delta y_{2}\right)\right]/\sqrt{\left(\Delta {x_{1}}^{2}+\Delta {y_{1}}^{2}\right)\left(\Delta {x_{2}}^{2}+\Delta {y_{2}}^{2}\right)} \end{array}$$

### Urban network system analysis

Based on the UCINET 6.0 software, the urban network system of GBA is analyzed by four aspects, including economic connectivity, network density, network centrality, and center-periphery (38, 39). Moreover, the values and characteristics of its network structure are examined and visualized using GIS.

#### Economic connectivity degree

The regional economic linkage can measure the regional economic intensity, and the modified gravity model is employed to measure the regional economic linkage. The matrix is adopted to analyze the network model, involving the indicators mainly including the regional GDP, resident population, and the shortest path time of the road obtained through modified gravity of time distance:


(4) $$\begin{array}{l} R\text{ij}=\left(\sqrt{P\text{i}G\text{i}}\times \sqrt{P\text{j}G\text{j}}\right)/D^{2}\text{ij} \end{array}$$

where *R*_*ij*_denotes the intensity of economic linkage between regions *I* and *j, Pi* and *Pj* represent the number of populations in areas *i* and *j, Gi* and *Gj* express the acquired GDP in areas *i* and *j*, and *D*_*ij*_ is the time of the shortest path based on the road network between two regions, *i* and *j*.

#### Network density

Network density is an essential indicator for analyzing the closeness of organizational relationships among nodes in a network. Network density is expressed as the ratio of the number of connections between city nodes in the network to the number of relationships in theory. The higher the value of network density, the stronger the connections between node members will be. Subsequently, network density can be expressed as follows:


(5) $$\begin{array}{l} D_{ij}=\overset{K}{\underset{i=1}{\sum }}\overset{K}{\underset{j=1}{\sum }}d\left(C_{i},C_{j}\right)/k\left(k-1\right) \end{array}$$

*D*_*ij*_ denotes the network density; *d*(*C*_*i*_*, C*_*j*_) represents the number of connections between cities *I* and *j*; *k* expresses the number of city nodes.

#### Network centrality

Network centrality is generally divided into three measures, namely, point centrality, proximity centrality, and intermediate centrality. In this study, the point of centrality is selected since it measures the centrality of a node city in the network and reflects the city's ability to control resources and markets. The higher the value, the stronger the core competitiveness of the city will be. The point centrality formula is expressed as follows:


(6) $$\begin{array}{l} C_{Di}=\overset{K}{\underset{i=1}{\sum }}Xij/\left(k-1\right)\left(i\neq j\right) \end{array}$$

where *C*_*Di*_ denotes the point degree centrality of a city, *X*_*ij*_ is the amount of connection between two cities, and *k* is the number of city nodes.

#### Center-periphery analysis

The center-periphery structure can be quantitatively analyzed according to the closeness of the connection between nodes in the network. The network “location” structure can be quantitatively analyzed to distinguish the core and edge of the network. If cities are interconnected and frequently interact in terms of information sharing and economic cooperation, they can form a cohesive subgroup, while the cities are sparsely or unconnected and do not constitute a cohesive subgroup. Through the cohesive subgroup, the state of the internal substructure of urban agglomeration can be revealed and characterized, and the development status of the urban network can be obtained from a macro perspective. Furthermore, the nodes in the network are assigned to two regions, including the core area and the periphery area, and the nodes in the core area take up a more important position in the network.

## Study areas and data sources

### Study areas

**Figure 2:**
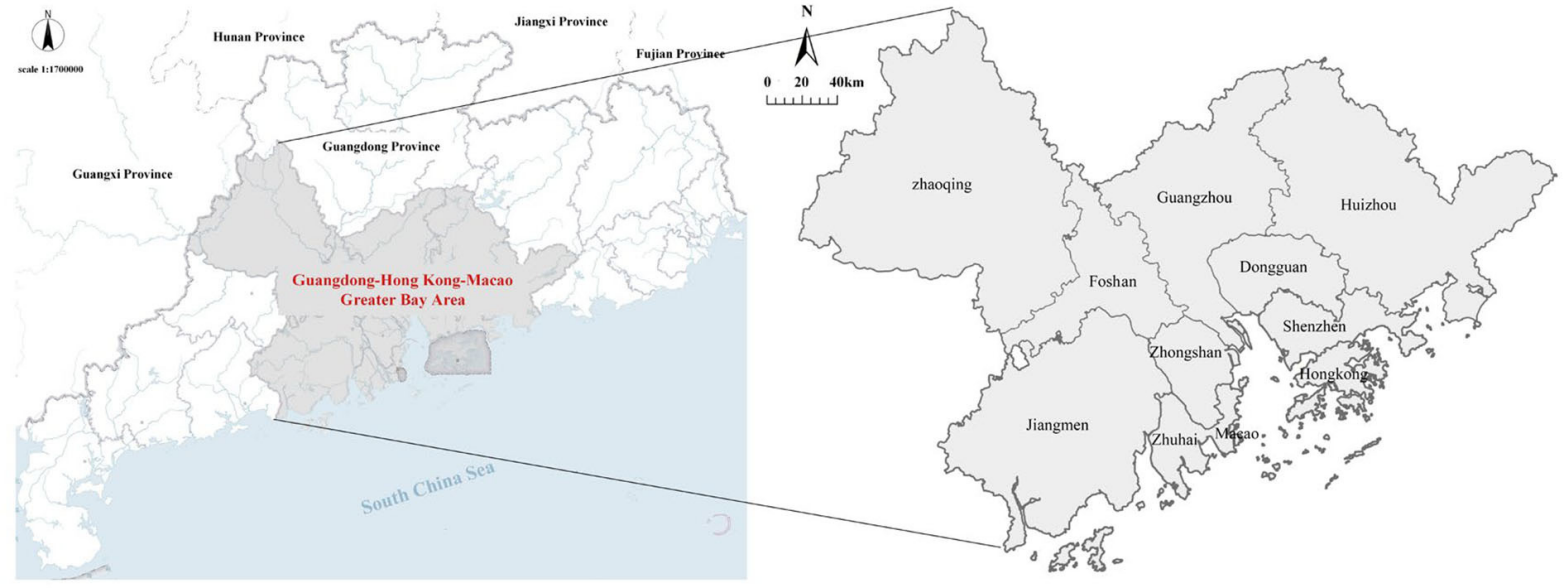
Location of the GBA.

The GBA includes Guangzhou, Shenzhen, Foshan, Dongguan, Zhuhai, Huizhou, Zhongshan, Jiangmen, Zhaoqing, and the Hong Kong and Macao special administrative regions, as listed in Figure 2. The GBA is densely populated and highly urbanized. It shows an industrial structure dominated by an export-oriented economy, with import and export trades accounting for nearly 1/3 of the country, making it a novel platform and a virtual space for the government to build a higher level of international economic, trade, scientific, and technological cooperation and innovation development. At present, the economy of the GBA is being re-transformed and upgraded, constantly developing in the direction of technology-intensive, operation-intensive, and urban-rural integration, which leads to the formation of an intelligent, modernized, internationalized, and shared economic system.

### Data sources

The data sources consist of Guangdong Statistical Yearbook, Guangzhou Statistical Yearbook, Foshan Statistical Yearbook, Shenzhen Statistical Yearbook, Zhuhai Statistical Yearbook, Huizhou Statistical Yearbook, Jiangmen Statistical Yearbook, Dongguan Statistical Yearbook, Zhaoqing Statistical Yearbook, and Zhongshan Statistical Yearbook between 2011 and 2021 in the PRD, and Macao Statistical Yearbook and Hong Kong Statistical Yearbook between 2010 and 2020 (note that the statistical yearbooks of Hong Kong and Macao are all data of the current year). Due to the different statistical standards of each city, the data taken in this study are those of the unemployed population in Hong Kong and Macau for the number of registered urban unemployed, total retail sales of consumer goods in Hong Kong, total retail sales in Macau, and total trade in Hong Kong and Macau for whole trade import and export. All of the above are converted to RMB value in accordance with the exchange rate of the year.

## Results

### Economic pattern

#### Economic gravity center

**Figure 3:**
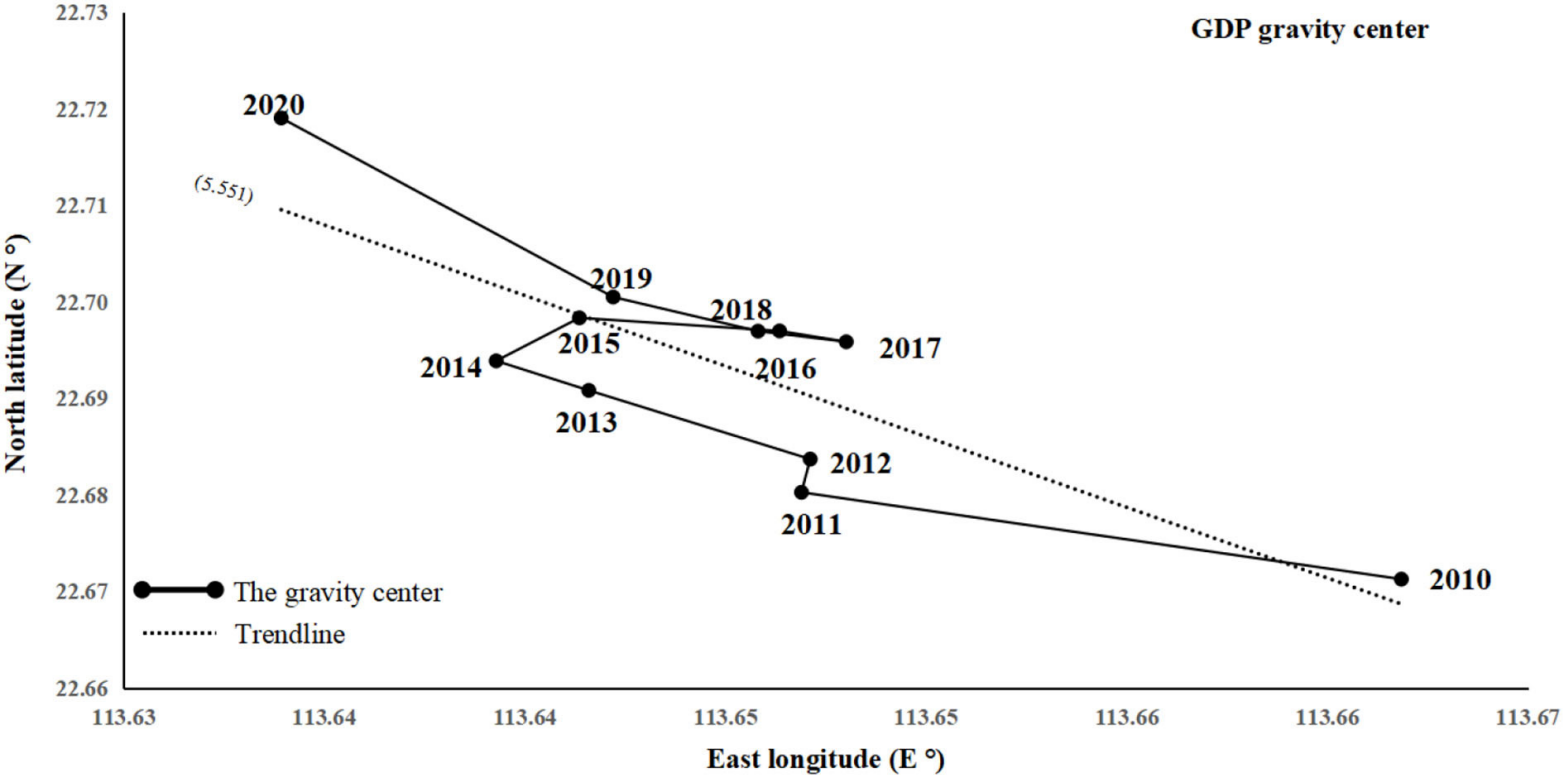
Movement in the economic gravity center of the GBA.

The center of gravity and the distance of movement of the center of gravity of the GDP of the GBA from 2010 to 2020 are examined using the center of gravity formula, as illustrated in Figure 3. ① For distribution location, the center of gravity of the economic resilience of the Greater Bay Area in the decade is located near 113.64° E, 22.69° N, i.e., in Nansha district, Guangzhou. ② The direction of the center of gravity shifts in northwest-east-northwest order, with an overall change to the northwest over the past decade. The main reason for the directional change of the center of gravity is that Zhuhai, relying on the special economic zone, policy support, and other advantages, has seized the opportunity for economic development and optimized the macroeconomic environment. Its high-tech industries have been developed, and its weight of GDP surpassed Jiangmen and Zhongshan at once after 2018 such that the western economy has been progressively boosted. Besides, Hong Kong's economy began to go down after the recession arising from the international financial crisis, which weakened the overall economic development level of the East Wing to a certain extent. ③ From the perspective of the center of gravity movement, the gravity center of economy moved the most in 2010–2011, followed by 2019–2020. The least was in 2016–2017, with a cumulative movement of 5.551 km over the past decade, and the overall movement rate shows the “large-medium–large” characteristics. In 2011, as China was still in the stage of high-speed economic development, cities (e.g., Foshan, Shenzhen, Guangzhou, and Macau) ushered in rapid development. Still, Hong Kong was subjected to a financial crisis at this time, and the economic growth rate was not as fast as before. The economic center of gravity did not shift much between 2016 and 2017 mainly because the total GDP of Guangzhou and Shenzhen was in a balanced state, with Guangzhou's GDP above Shenzhen's in 2016 and Shenzhen's GDP overtaking Guangzhou's in 2017. With the pull of the two economies and the stable growth of each city, the economic center of gravity of the GBA also tended to stabilize. After the COVID-19 pandemic, most cities underwent economic stagnation or slowed down (e.g., Dongguan, Foshan, Zhuhai, and Shenzhen) such that Hong Kong and Macau experienced a severe economic decline. This public health event has significantly declined the GDP growth rate of the GBA. However, Guangzhou's economic growth was boosted after the COVID-19 pandemic such that the gravity center increased significantly and gradually reversed to the northwest between 2019 and 2020.

#### Economic growth factor gravity center

**Figure 4:**
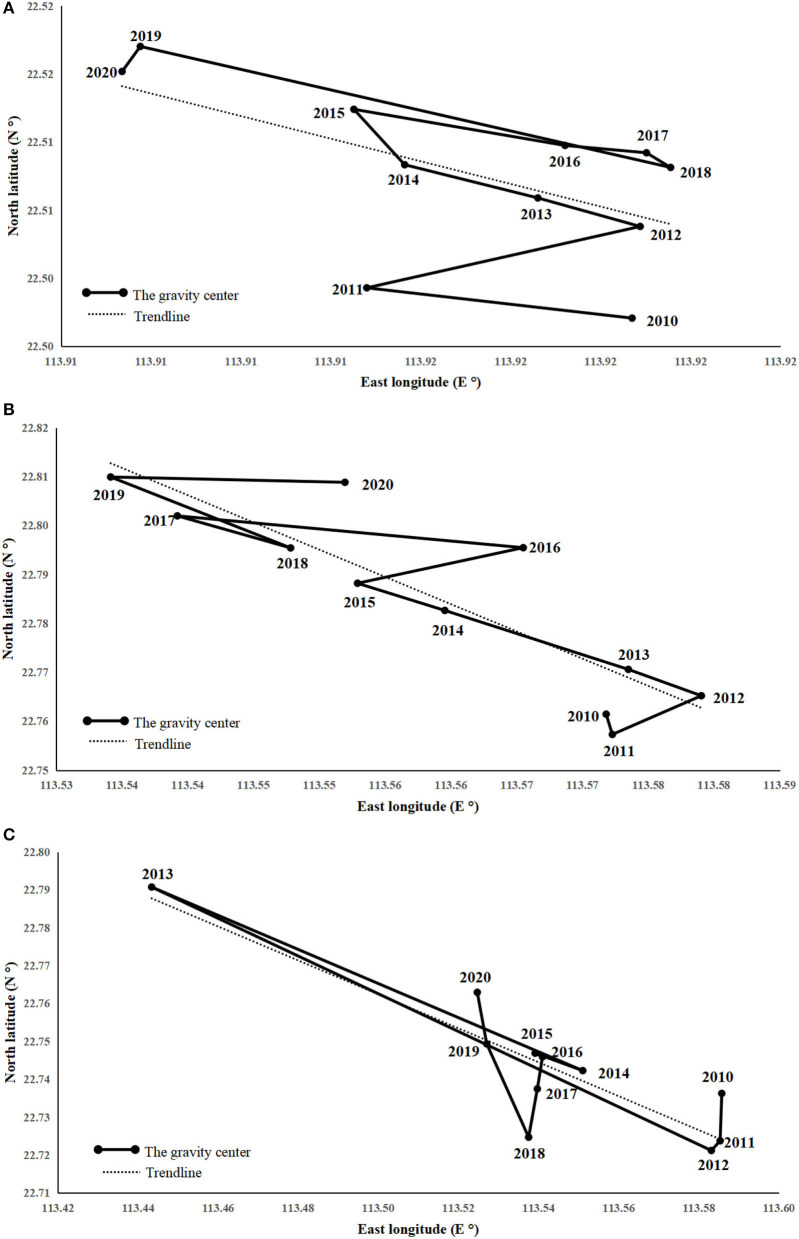
Movement in the gravity center of **(A)** imports and exports, **(B)** consumption, and **(C)** investment.

The change of the economy's gravity center only reveals the economic pattern from an overall perspective, which should be explored in-depth based on the factors of the economy's center of gravity. The three elements (i.e., import and export, consumption, and investment) are selected for the center of gravity analysis. As depicted in Figure 4, the center of gravity of all the three factors shifted from southeast to northwest. To be specific, the center of gravity of import and export showed a “zigzag migration” to the northwest, to the northeast, to the northwest-northeast, and finally to the northwest, with an overall shift of 4.146 km. The most significant change was between 2018 and 2019. The reason for the above result is that the total trade volume of Hong Kong exceeds more than half of that of other cities in the Bay Area, and its trade status is recognized to be critical. However, the trade volume plummeted after 2018, and the growth rate of the other three major trade cities in the central-eastern part of the Bay Area (Shenzhen, Dongguan, and Guangzhou) has flattened. The center of gravity of consumption showed a “zigzag migration,” whereas the overall deviation was 14.563 km, and the most significant change was between 2016 and 2017 due to the surge in social retail sales in Foshan and Shenzhen after 2016. As a result, the east-west part of the Bay Area has shifted back and forth .The investment center of gravity displays an “irregular migration,” first migrating due south, then to the northwest, then offset southeast-due south, and finally folding to the northwest. The overall offset was 36.006 km, and the most significant compensation was between 2012 and 2013. The reason for the above result is that there was a surge and a plunge within 2 years after Zhaoqing fixed asset investment between 2012 and 2014, whereas Shenzhen and Foshan began to soar. However, in 2018, there was a slowdown in the growth of Foshan, Hong Kong plunged, and Guangzhou soared in parallel.

### City network system

#### Economic connectivity degree

**Figure 5:**
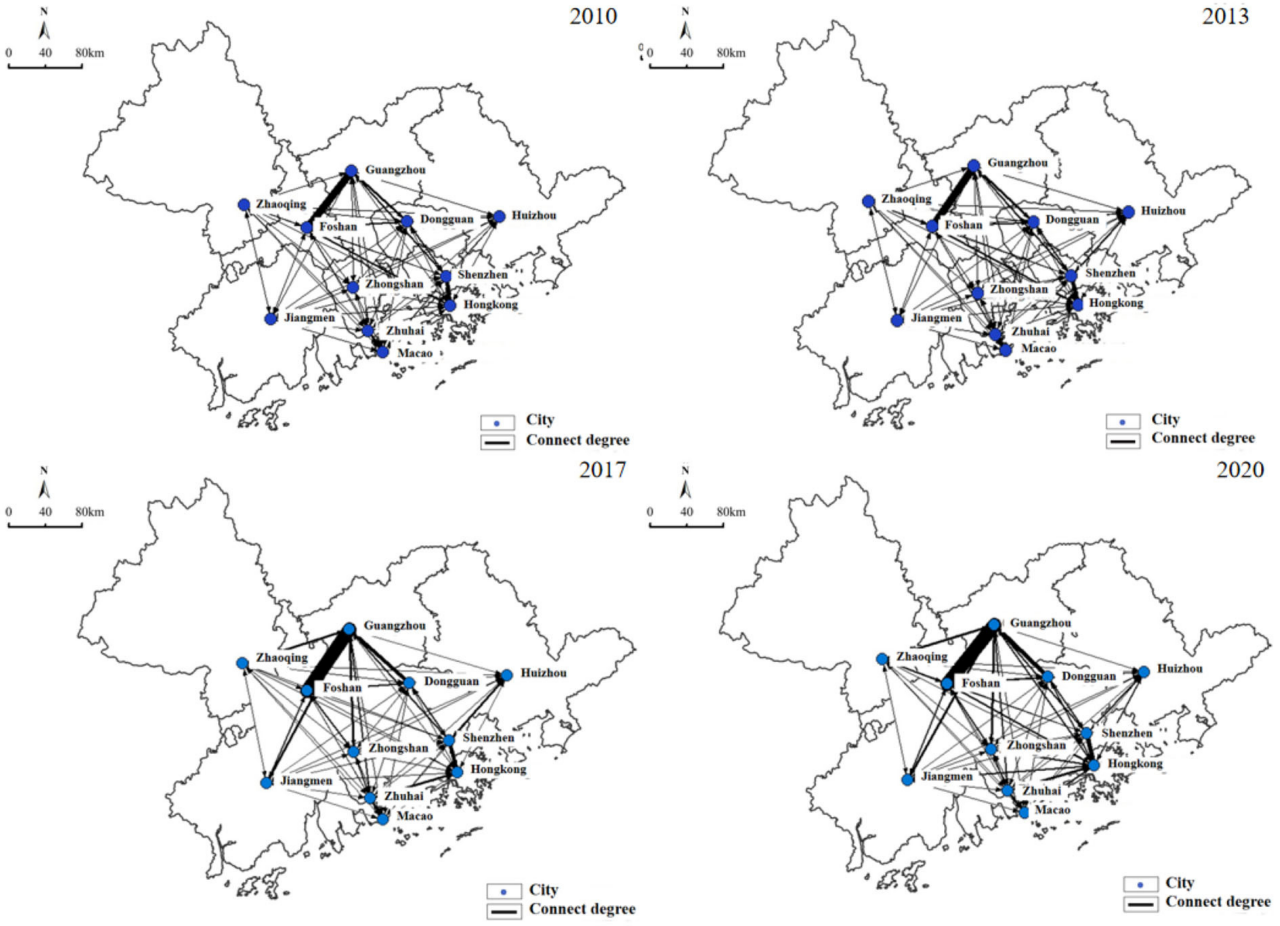
Economic connectivity of the GBA.

Economic relationships and interactions have critical significance in the balanced development of the entire region with each other. As depicted in Figure 5, the economic linkage of the GBA from 2010 to 2020 was rising fast, and the linkage of Guangzhou-Foshan occupying the core position showed a trend of extending from the central city to the peripheral cities. The top 3 economic linkages in 2010 included the linkage of Guangzhou-Foshan (27,168.442), the linkage of Foshan-Guangzhou (14,479.068), the linkage of Hong Kong-Shenzhen (8,172.332), and the last one was the linkage of Huizhou-Macao (5.536). The above results suggest that the economic linkage of Guangdong-Hong Kong-Macao Greater Bay Area cities has been primarily revolved around the top in 2013. The total economic linkage of GBA has increased stably, and the number of links that reached the average level of economic linkage increased by 5 compared with 2010, among which the relations between Guangzhou, Foshan, Hong Kong, and Shenzhen still increased significantly, with the linkage over 8,000. Huizhou-Macao still ranked last (8.425), thus suggesting the significant gap between the economic linkages in the GBA. The overall economic relations display an unbalanced trend. Thirty-five linkages reached the average standard of the total and economic relations in the GBA in 2017, and the network was kept balanced. To be specific, the economic linkage of Guangzhou-Dongguan (15,475.101) surpassed that of Hong Kong-Shenzhen (13,320.92) in the forefront, suggesting that the core position of Guangzhou in the GBA is enhanced. The economic linkage has boosted the surrounding cities (e.g., Zhaoqing, Zhongshan, Zhuhai, and other cities on the west bank of the Pearl River Estuary). The growth of economic linkages in the Guangdong-Hong Kong-Macao Greater Bay Area slowed down in 2020, primarily arising from the effect of the COVID-19 pandemic. The economic linkages among the cities continue to grow stably.

#### Urban network density

**Figure 6:**
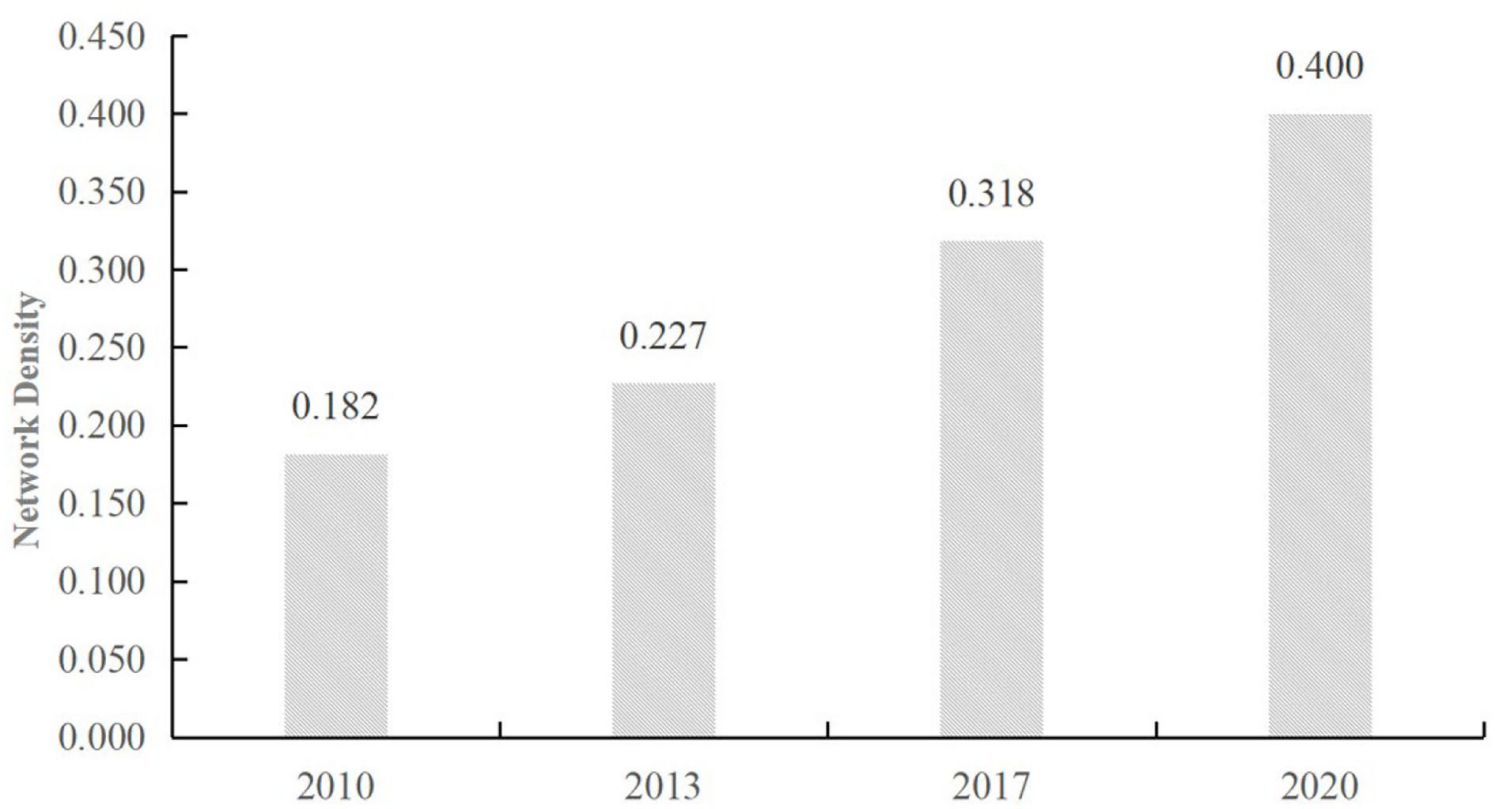
Analysis of network density in the GBA.

Network density indicates the tightness of connections among the network members. The greater the density of network ties, the tighter the economic relations among cities will be, and the greater number of channels of the economic relations and cooperative behaviors among cities will be. As depicted in Figure 6, the network density of cities in the GBA reached .182 in 2010, thus suggesting that the path of core cities is dependent on other cities. Moreover, the edge cities are subjected to greater geographical and distance constraints, and these cities achieve a poor degree of network connection. With the growth rate of economic development in the Pearl River Delta (PRD) and the sense of internal collaboration, the density was 0.2273 in 2013 as compared with 2010, marking a growth rate of .25, whereas the growth is not significant. With 2013 as the turning point, it is significant that the overall network density grew more in 2017, reaching 0.3182 with a growth rate of 0.4, thus suggesting that the connection between cities in the GBA has been deepened significantly. The spatial network density increases steadily, and the economic connection between the respective nodes is obvious along with the framework agreement on deepening cooperation among Guangdong, Hong Kong, and Macao to promote the construction of the Greater Bay Area. The economic network density was 0.4 in 2020 due to the COVID-19 pandemic. However, the network density growth rate of the Greater Bay Area has declined. The network density of the Greater Bay Area still maintains a growing trend because of the development foundation in 2018 and 2019.

#### Network centrality

**Figure 7:**
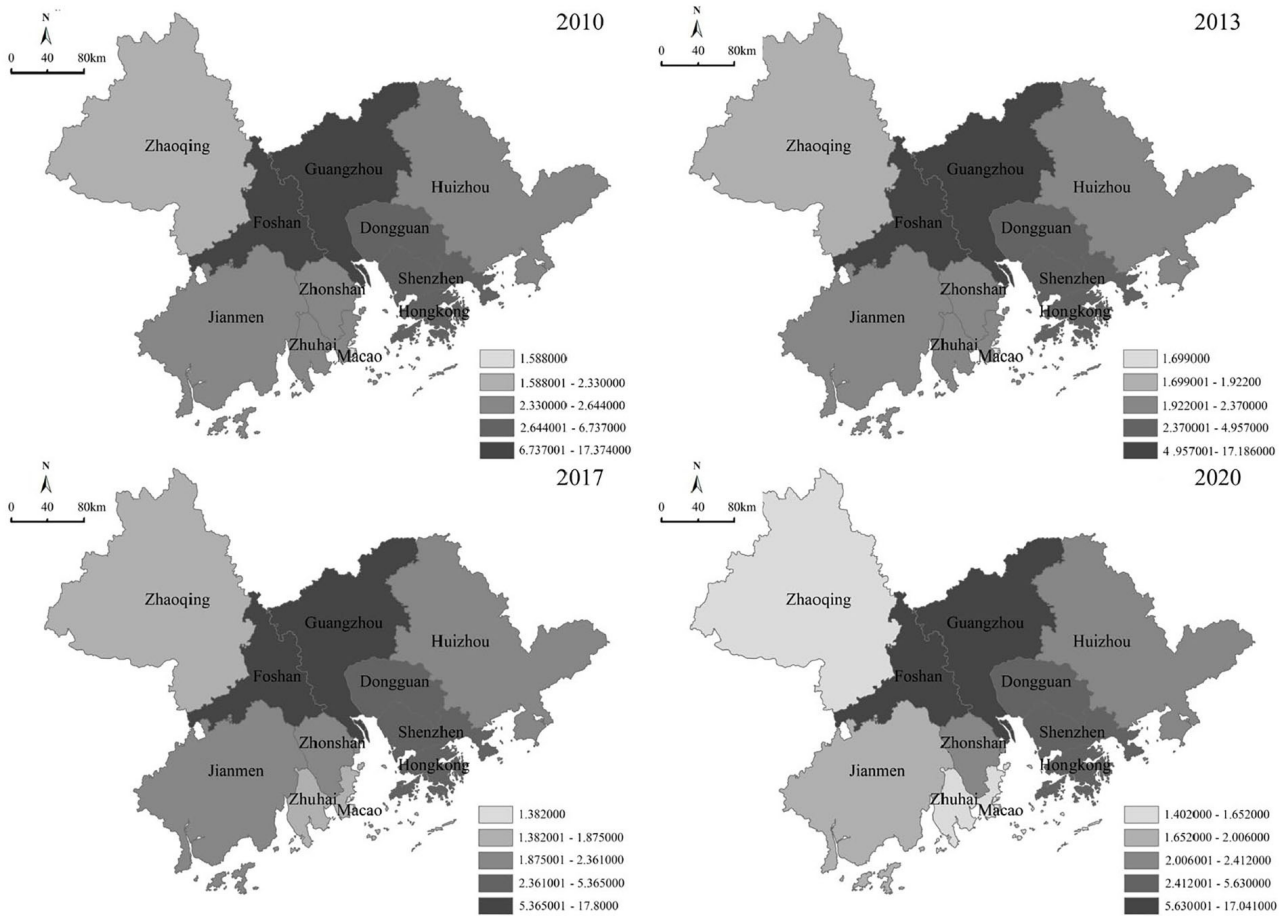
Network centrality of the GBA.

Network centrality is capable of measuring the centrality of an entire network and indicating the degree of integration and consistency of a whole network system. The network centrality in the GBA tends to increase, whereas its internal unevenness leads to a prominent polarization feature. As listed in Table 1 and Figure 7, the point degree centrality of cities close to the Pearl River Estuary (e.g., Guangzhou, Foshan, and Hong Kong) was generally high in 2010, while the peripheral cities were in a relatively weak situation. Guangzhou, the administrative center of Guangdong province and the core city of the GBA, has consistently maintained a high level of node centrality. Coupled with the optimization of industrial structure and convenient transportation facilities, Guangzhou's economic connectivity and importance in the GBA have been increasing over the past few years. Shenzhen's node centrality decreased in 2013, whereas the value is rising, thus revealing that the traditional Pearl River Delta urban economic network began to integrate among the cities in the peripheral nodes, and a balance is formed between the cities on both sides of the Pearl River Estuary. In 2017, the point-degree centrality of Dongguan, Zhongshan, and Zhuhai was more prominent, suggesting that the radiation effect of the Greater Bay Area was significantly improved, and the main economic linkage channel was formed initially. In 2020, affected by the COVID-19 pandemic, the point degree centrality values of Zhuhai and Shenzhen, two cities close to Hong Kong and Macao, significantly decreased, while the point degree centrality of Huizhou, Zhaoqing, Jiangmen, and other peripheral cities increased, suggesting that the polarization of economic linkages in the GBA eased and that the network centrality started to outbalance. In general, the core cities of the PRD (e.g., Guangzhou, Shenzhen, Hong Kong, and Foshan) have critical significance in increasing the flow of factors and the optimal allocation of resources because of their better economic foundation and more robust capacity for industrial interaction and collaboration. In contrast, the peripheral cities (e.g., Zhaoqing and Huizhou) exhibit a lower network centrality because of farther transportation distance and weaker economic foundation. Macau has been less centralized because of its relatively homogeneous industrial structure and institutional policies.

**Table 1:** Node centrality of the GBA.

	**2010**		**2013**		**2017**		**2020**	
**City**	**Centrality**	**Ratio**	**Centrality**	**Ratio**	**Centrality**	**Ratio**	**Centrality**	**Ratio**
Guangzhou	17.374	0.289	17.186	0.308	17.178	0.312	17.041	0.317
Foshan	12.325	0.205	11.900	0.213	11.785	0.214	11.683	0.217
Shenzhen	5.685	0.095	4.957	0.089	5.365	0.097	5.630	0.105
Hong Kong	6.737	0.112	4.723	0.085	4.482	0.081	3.808	0.071
Dongguan	4.538	0.076	4.318	0.077	4.260	0.077	4.262	0.079
Zhuhai	2.330	0.039	2.252	0.040	1.826	0.033	1.625	0.030
Zhongshan	2.644	0.042	2.370	0.043	2.361	0.043	2.412	0.045
Jiangmen	2.525	0.038	2.288	0.041	2.213	0.040	2.006	0.037
Macau	1.588	0.026	1.699	0.030	1.382	0.025	1.402	0.026
Huizhou	2.265	0.038	2.142	0.038	2.309	0.042	2.247	0.042
Zhaoqing	2.104	0.035	1.922	0.03	1.875	0.034	1.652	0.031

#### Center-periphery analysis

**Figure 8:**
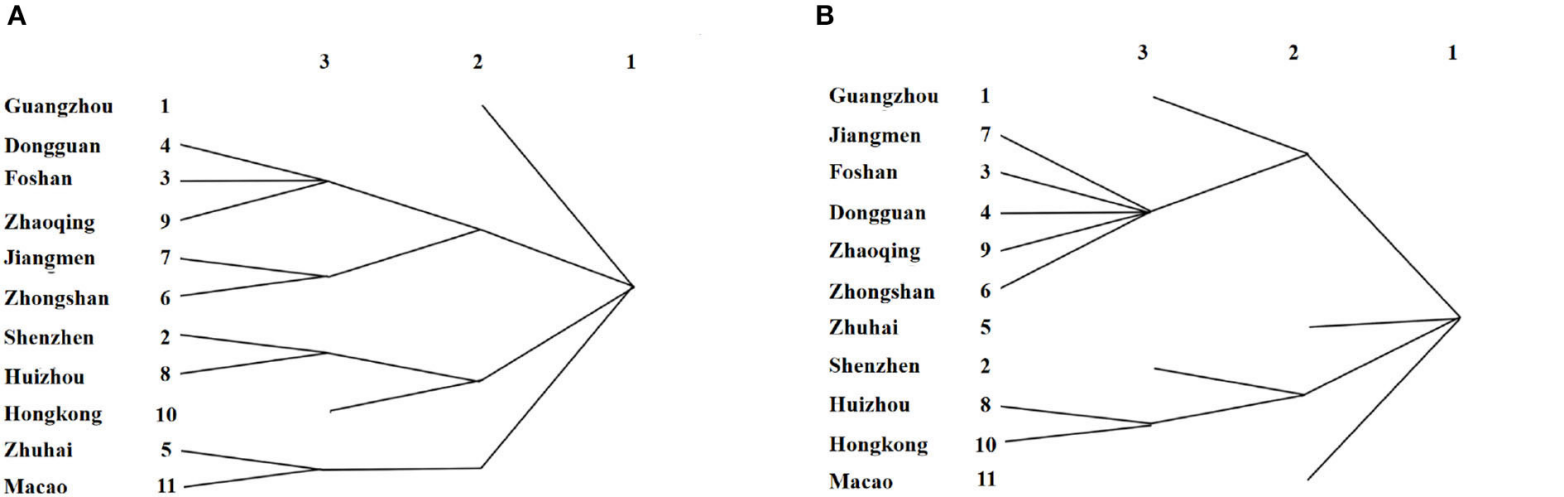
Cohesive subgroups of GBA in **(A)** 2010-2019 and **(B)** 2020.

In accordance with the center-periphery theory, the region is divided into core and peripheral areas, which indicate the internal differences and connections of the regional spatial system. Based on the economic linkage matrix of cities in the GBA, a non-overlapping cluster analysis is conducted based on the iterative correlation convergence method in the UCINET software to examine the division relationship of the cohesive subgroups of the network. The average value of the economic linkage in 2010 is adopted as the threshold for binarization to study the core-periphery structure of the urban system. As depicted in Figure 8, the membership composition of each subcluster is relatively stable from 2010 to 2019 and forms four major cohesive subclusters, Guangzhou, Shenzhen-Huizhou-HongKong, Zhuhai-Macao, and Dongguan-Foshan-Zhaoqing-Jiangmen-Zhongshan. Hong Kong and Macau, the major special economic zones in the Bay Area, can drive the development of neighboring cities (e.g., Huizhou and Zhuhai), and other cities in the PRD also have a stable cohesive effect. However, 2020 shows certain changes, with Guangzhou's linkage effect having a certain increase and the cohesive effect on several cities around the PRD enhancing, Moreover, the cohesive subclusters of Zhuhai and Macau show their differentiation under the effect of the COVID-19 pandemic and the international economic environment. The cohesive subcluster of Shenzhen-Hong Kong-Huizhou remains unchanged. In general, the clustering results of the subgroups remain consistent with the degree of geographic proximity and economic association of the cities and exhibit an inside-out circle pattern in space.

**Figure 9:**
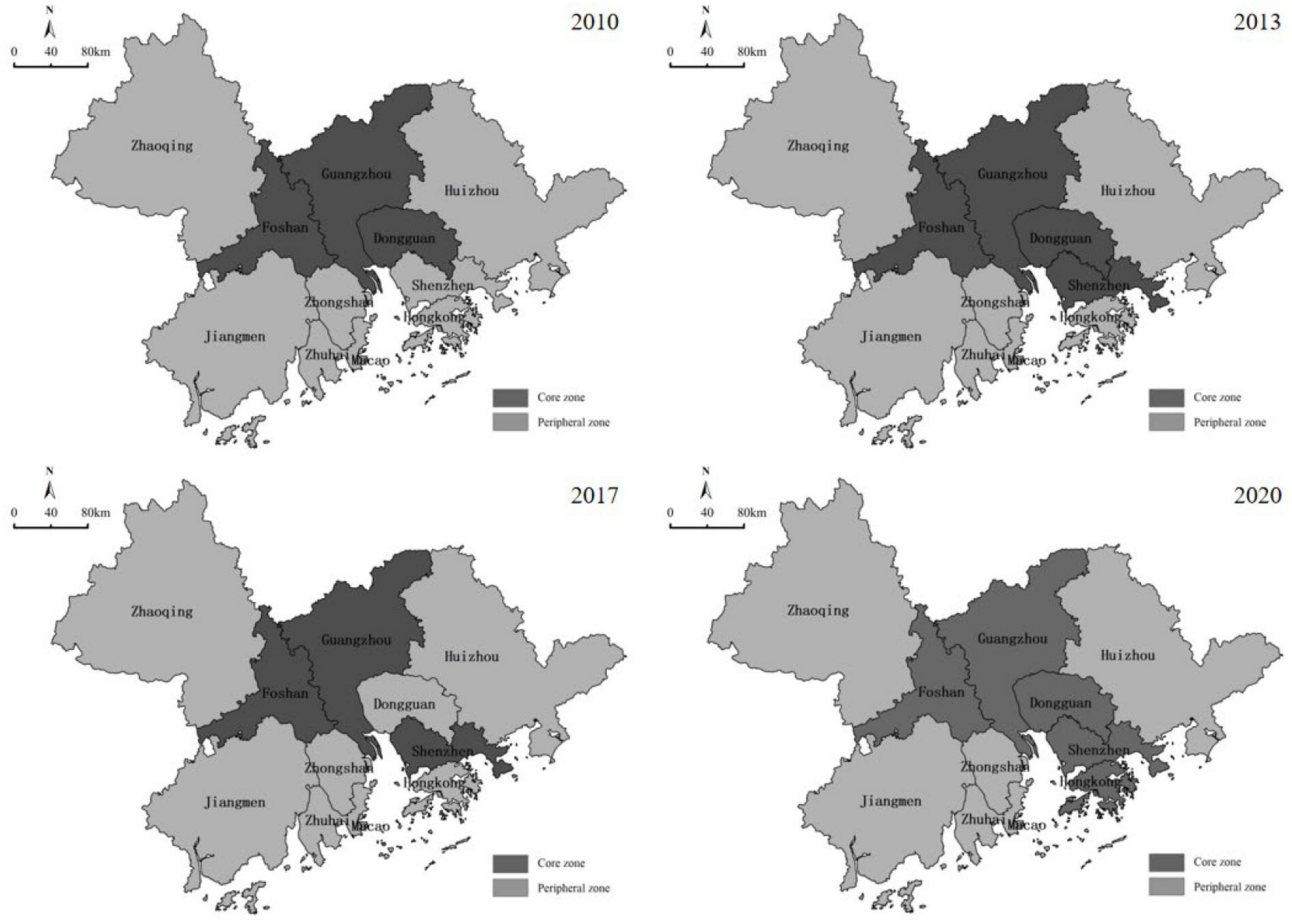
Core-periphery analysis of the GBA.

As depicted in Figure 9, the network core area of the GBA has spread from the traditional cities in the Pearl River Delta to the cities on the east coast of the Pearl River Estuary over the past decade. Moreover, an urban development axis and a core cluster of Guangzhou-Dongguan-Shenzhen-Hong Kong have emerged. In 2010, Guangzhou, Shenzhen, and Foshan belonged to the core area, subject to the factor of distance leading to the generation of solid linkages of network groups, whereas the efficiency of new economic information input has declined. Besides, institutional or traffic conditions have hindered Hong Kong, Macau, Zhaoqing, Huizhou, and other cities while belonging to the peripheral areas. However, in 2020, along with the rapid development of the “Shenzhen-Hong Kong-Guangzhou” innovation cluster and the further implementation of the development strategy of the GBA, the connection among Hong Kong and Shenzhen, Guangzhou, Dongguan, and other cities is progressively enhanced such that a core area has been formed on the east coast of the Pearl River. Furthermore, with the support of institutional advantages and innovation drive, it has become the core engine of the GBA. In brief, the core-edge structure of the GBA is more apparent, and the internal economic linkages can be deepened later.

## Conclusion, recommendations, and discussion

### Conclusion

(1) The economic gravity center of the GBA is moving northward as a whole with comparatively obvious phases in the direction and rate of movement. Because of the COVID-19 pandemic, the overall economic pattern, consumption, import and export, and investment in the GBA have seen certain changes in the opposite direction, which also fully illustrates the importance of preventing and controlling major public health events. The center of gravity of GDP in the GBA has been located in the Nansha district of Guangzhou over the past decade. It is still shifted in the northwest-east-northwest direction, and the movement rate exhibits the characteristic of “large-small-large.” The import gravity, export gravity, and consumption gravity centers show a “zigzag migration” in which import and export first move to the northwest, to the northeast, to the northwest-northeast, and finally to the northwest, with an overall shift of 4.146 km. The most significant change was between 2018 and 2019. The center of gravity of consumption moves in the opposite direction to the center of gravity of import and export, with an overall shift of 14.563 km. The center of gravity of consumption moves in the opposite direction to the center of gravity of import and export, with a general change of 14.563 km, and the most significant shift was between 2016 and 2017. Lastly, it shifts to the northwest, with an overall change of 36.006 km and with the most significant change being from 2012 to 2013.

(2) The city network of the GBA has been enhanced, and the spatial structure has been stabilized, but there is still an obvious “core-periphery” feature. From 2010 to 2020, the degree of economic linkage in the GBA rapidly increased, the economic network tended to mature, and the economic and spatial proximity effects of the region emerged. Guangzhou, Shenzhen, Foshan, and Dongguan initially established a backbone network of economic linkages and spread in a radial trend, and the overall economic network gradually rose over time with balanced economic relations. The analysis of the social network structure reveals that the network density of the GBA is steadily increasing, that the core nodes are path-dependent on other cities, and that the periphery cities have greater geographical and distance constraints. The four major cohesive subgroups are relatively stable. The core cities in the Bay Area are more central, while the economic cities in the edge areas are slightly less central, and the unevenness of network centrality within the region leads to the prominent polarization characteristics. The core-periphery structure of the GBA is more significant, and the internal connection and coordination are not deepened sufficiently. Furthermore, the core area of the network spreads from the traditional cities in the Pearl River Delta to the cities along the Pearl River Estuary. The connection among Hong Kong, Shenzhen, Guangzhou, and Dongguan has been progressively deepened, forming an important core area in the GBA. In brief, the spatial structure of economic linkages and urban networks in the GBA has changed under the influence of the COVID-19 pandemic, thus reshaping regional development clusters and synergistic development paths.

### Recommendations

(1) Optimizing the diversification of industrial structure and building a coupled development model of “import/export-consumption-investment”.

From the economic contraction-recovery process of each city during the financial crisis and the new epidemic, we can see that Guangzhou and Shenzhen, with diversified industrial structures and strong technological innovation, were less affected, while cities with traditional industries and single structures were more affected. With further globalization, the future development of cities will inevitably respond to changes in international conditions and industries, and overly homogeneous industries and markets will inevitably mean greater risks. Therefore, there is a need to promote a diversified industrial structure to achieve multi-point support and diversified development of the regional economy and enhance the synergistic effect of the GBA. For instance, the implementation of the “strong to drive weak” urban co-development strategy, Guangzhou and Foshan to drive Zhaoqing synergistic development, Shenzhen and Dongguan to promote the mutual development of Huizhou, Zhuhai, and Zhongshan to drive the win-win development of Jiangmen, Hong Kong, and Macao to create a special economic road, form the “three metropolitan areas + two special administrative regions” development pattern. Under a complex economic environment abroad and the disruption of public health events, it can effectively decompose and cache external risks, increase the stability of the economic system of GBA, and establish a more resilient, healthy, and coordinated regional economic system by building a coupled development model of “import/export-consumption-investment”.

(2) Building sub-level hub cities to achieve multi-point support and synergistic development of the regional economy.

For spatial structure, the center of GBA is significantly polarized. Notably, the development of cities in the deep interior and the mouth of the west bank is relatively lagging, and the imbalance of urban development hinders the development of the Bay Area as a whole. The future development should follow the perspective of an urban network, cultivating secondary hub cities on the inland and the west bank of the Pearl River Estuary. Besides, the focus of the future development should be placed on the development of edge cities and promoting the interaction of industrial cooperation and park cooperation among cities of different levels. Building a city system with graded levels can enhance the spatial connectivity of the whole city cluster and make it easier to affect the core cities while facilitating the coordination and integration of the whole city cluster, increasing the stability of the economic system of the GBA, and forming a more resilient, healthy, and coordinated regional economic system.

(3) Building a network of cities in the GBA with “complementary advantages, staggering development, and circular interoperability”.

For the characteristics of the spatial structure of the “core-periphery” urban network, it is necessary to build a network of cities in the GBA with “complementary advantages, staggering development, and circular interoperability.” On the one hand, GBA should actively play the role of organization and drive core cities (e.g., Hong Kong, Shenzhen, and Guangzhou); create three regional growth poles: Hong Kong-Shenzhen, Guangzhou-Foshan, and Macau-Zhuhai. The strong combination of the three growth poles plays a leading role to enhance the overall strength. Moreover, GBA should gradually narrow the gap between core cities and neighboring cities through the rational deployment of resources, the industrial division of labor, regional support mechanism, and improvement of the transportation network, facilitating the concentration and flow of production factors (e.g., products, talents, services, and capital in the region), and form a high-quality urban network system of “innovation, green, openness, and sharing”, which are imperative for GBA governance after the COVID-19 pandemic.

### Discussion

The integration of Guangdong, Hong Kong, and Macao under the normalization of epidemic prevention and control has become a topic of concern for the future. The spatial structure of a regional economy plays a critical role in the development quality and sustainable development of a region. Based on the center of gravity model, gravitational force model, and social network analysis, the spatial elements of “points, lines, and networks” are adopted to explore the evolution of the economic pattern and urban network system of the GBA over the past decade, which complements the existing studies on regional economic structure and urban design from different research perspectives and methods. Moreover, the results of this study also illustrate the importance and effect of major public health events on economic gravity and urban network system while providing economic policy analysis for the government of the GBA to respond to the effect of the COVID-19 pandemic. However, the effect of public health events on regional economy is a complex process and is different from previous shocks triggered by finance, debt, economic cycles, etc. This study has some limitations. On the one hand, the long series evolution analysis and multifactor formation mechanism in the economic center should be further deepened. It is necessary to improve gravity model performance to changes in the level of aggregation of data and the temporal and spatial scale of economic patterns, urban mobility networks. Besides, limitations remain in socio-economic impact analysis, mechanism analysis, and forecasting (43, 44). Accordingly, novel urban mobility network models (flow space theory) or machine learning approaches are urgently required to more effectively predict fine-scale and high temporal-resolution urban mobility networks in subsequent research. On the other hand, data regarding people flow, logistics, traffic flow, information flow, and capital flow in the GBA were not included due to time constraints and data platform limitations, thus causing insufficient data accuracy and city network analysis still at the municipal level. Future research will be able to further clarify the driving forces and development schemes of the urban network system in accordance with the development path, public health events risk prevention, and governance policy strategies of the GBA, integrating social, cultural, and institutional factors actively, which provides guidelines for comprehensive economic competitiveness and healthy development.

## Data availability statement

The original contributions presented in the study are included in the article/supplementary material, further inquiries can be directed to the corresponding author/s.

## Author contributions

BT and YZ: conceptualization and funding acquisition. BT, ZC, YZ, and HS: methodology and writing—review and editing. ZC and HS: data curation. BT and ZC: writing—original draft and preparation. All authors have read and agreed to the published version of the manuscript.

## Funding

This research was funded by project of the 14th five-year plan for the development of Philosophy and Social Sciences in Guangzhou in 2021 (Grant No. 2021GZYB22); Scientific Research Project of Guangzhou Xinhua University (Grant No. 2018KYQN001); Project of Special Innovation Classes for Regular Universities in Guangdong Province (Grant No. 2020WTSCX136); Human Geography and Urban-Rural Planning, the Construction of Guangdong First-class Undergraduate Program (Grant No. F22MJ04JY).

## Conflict of interest

The authors declare that the research was conducted in the absence of any commercial or financial relationships that could be construed as a potential conflict of interest.

## Publisher's note

All claims expressed in this article are solely those of the authors and do not necessarily represent those of their affiliated organizations, or those of the publisher, the editors and the reviewers. Any product that may be evaluated in this article, or claim that may be made by its manufacturer, is not guaranteed or endorsed by the publisher.

## References

[1] Li M Ren B. Comprehensive evaluation of China's high-quality development in the new era and its path selection. Finance Eco Sci. (2019) 2019:26–40.

[2] Alokan OO. Globalization, inequality and the regional problem. Nig J Eco Soc Studies. (2006) 48:130–45.

[3] Ma G Gan G. Advances in spatial studies of regional economic development. Adv Geograph Sci. (2005) 2005:90–9.

[4] Lu Y. Theoretical refinement and regular recognition of spatial patterns in China. Acta Geographica Sinica. (2021) 76:2885–97. 10.11821/dlxb202112002

[5] Charlesworth E. A local example of the factors influencing industrial location. Geogr J. (1938) 91:340–51. 10.2307/1788189

[6] Dieleman FM Jobse RB. Aneconomicspatialstructureofamsterdam. Tijdschriftvooreconomischeensociale Geografie. (1974) 65:351–67. 10.1111/j.1467-9663.1974.tb01240.x

[7] James BK. Economic geography—spatial and environmental aspects of economic activity. Eco Geog. (2016) 2016:215–7. 10.2307/143804

[8] Guo T Xu Y Ma G Wang Z. A review of the theory and methods of regional economic spatial structure. Adv Geograph Sci. (2009) 28:111–8. 10.1007/978-1-4020-9623-5_5

[9] Ma L Liu Y. A review of research on the evolution of regional economic spatial structure under economic globalization. Adv Earth Sci. (2003) 2003:270–6.

[10] Lu Y. Study of Spatial Structure in Regional Development. Nanjing: Nanjing Normal University Press (1998).

[11] Liu A Yang K Xie X. A comparative study of new economic geography and traditional economic geography. Adv Earth Sci. (2005) 2005:1059–66. 10.3321/j.issn:1001-8166.2005.10.003

[12] Tóth KN. The changing economic spatial structure of Europe. Norw J Geog. (2014) 68:301–9 10.1080/00291951.2014.963665

[13] Niu F Wang F. Economic spatial structure in China: evidence from railway transport network. Land. (2022) 11:61. 10.3390/land11010061

[14] Nian M Sun J. Study on the change of regional economic spatial structure in China. Eco Theory Eco Manag. (2012) 2012:89–96. 10.3969/j.issn.1000-596X.2012.02.011

[15] Pang Y. Optimization of Regional Spatial Structure and Coordinated Regional Development in China. Wuhan: Wuhan University (2018).

[16] Ke W Lu Y Yu Z Wang H Ma Y. Multivariate driven spatial pattern evolution of Jiangsu county economy. Acta Geographica Sinica. (2013) 68:802–12.

[17] Li X Qiao J. Spatial analysis of inter-county economic differences in China in the 1990s. Acta Geographica Sinica. (2001) 2001:136-145. 10.11821/xb200102002

[18] Jing C. Geographical scale, industrial diversity, and regional economic stability. Growth Change. (2019) 50:609–33. 10.1111/grow.12287

[19] Aura R Pietro B Giovanni R Anette H Peter N. Regional labour markets and job accessibility in City network systems in Germany. J Trans Geography. (2010) 19:528–36. 10.1016/j.jtrangeo.2010.05.008

[20] Fu Y Zhong Y Feng X. Spatial structure evolution of regional economy in yangtze river economic belt. World Geography Res. (2018) 27:65–75. 10.3969/j.issn.1004-9479.2018.03.007

[21] Sung TP Byeoung CJ. Analysis of regional economic structure and regional input-output using RW and LQ methodology. Journal of Industrial Economics and Business. (2016) 29:541–62.

[22] Dou J Zhang K. Evolutionary trends of regional economic patterns and urban network systems in China. Urban Issues. (2015) 2015:54–61.

[23] Güneş K Benjamin W. Global factors and trend inflation. J Int Econ. (2020) 122:103265. 10.1016/j.jinteco.2019.103265

[24] Yang Z Chen Y Zhang P. Macroeconomic shocks, financial risk transmission and governance responses under major public emergencies. Manag World. (2020) 36:13–35+7.

[25] Zhou M. Economic center of gravity. regional disparities and coordinated development, China Social Science. (2000) 20:42–53.

[26] Ding H Li P. Analysis of the trajectory of population and employment centers in guangdong province since the founding of the people's republic of China. Industrial Technology and Economics. (2009) 28:79–84. 10.3969/j.issn.1004-910X.2009.04.021

[27] Yang J Lei S Wang G. Analysis of the evolutionary path and offset of the center of gravity of wheat production. Chin Agric Sci Bull. (2008) 24:504–9.

[28] Li Q Ren Z Zhang L. Analysis of the spatial evolution trajectory of China's railroad transportation center of gravity in the past 30 years. Arid Zone Geography. (2009) 32:119–24.

[29] Huang J Feng Z. The evolutionary path of socio-economic center of gravity and environmental pollution center of gravity in Shaanxi Province and its comparative analysis. Human Geography. (2006) 21:117–22. 10.3969/j.issn.1003-2398.2006.04.025

[30] Wan S Chen S Shen Z. The law of shifting tourism focus in six central provinces and implications for tourism cooperation. Areal Res Dev. (2010) 29:91–4. 10.3969/j.issn.1003-2363.2010.02.018

[31] Carmen BP. Urban Networking vs. Smart City. J Busin Public Admini. (2017) 8:73–86. 10.1515/hjbpa-2017-0006

[32] Sun Y Yao S Zhang L. Spatial expansion of urban network for the three coastal agglomerations of China: a study based on integrated traffic information network. Sci Geographica Sinica. (2018) 38:827–37.

[33] Tang C Ma X. Spatial pattern and structure of networked logistics links in Chinese cities–a study based on data of express delivery outlets. Prog Geography. (2020) 39:1809–21. 10.18306/dlkxjz.2020.11.003

[34] Pan F Fang C Li X. The progress and prospect of research on Chinese City network. Sci Geographica Sinica. (2019) 39:1093–101.

[35] Wu D Zhu Q. A preliminary study on quantitative regional classification methods. J Beijing Normal Univ. (2003) 39:412–6. 10.3321/j.issn:0476-0301.2003.03.023

[36] Wu C Liu J Gan G. Modern Economic Geography. Nanjing: Jiangsu Education Press (1997).

[37] Hu A Liu Y. Drift of the regional economic center of gravity and equilibrium trend in China. Eco Theory Eco Manag. (2013) 2013:101–9. 10.3969/j.issn.1000-596X.2013.12.010

[38] Zhong Y Feng Xi Wen Y. The evolvement and driving mechanism of economic network structure in the Changjiang river economic zone. Sci Geographica Sinica. (2016) 36:10–9. 10.1329/j.cnki.sgs.2016.01.002

[39] Peng F. Economic spatial linkages and spatial structure of Guangdong, Hong Kong, Macao and neighboring cities in the Greater Bay Area - an empirical analysis based on improved gravity model and social network analysis. Eco Geography. (2017) 37:57–64. 10.15957/j.cnki.jjdl.2017.12.008

[40] Amos OM. Unbalanced regional growth and regional income inequality in the latter stages of development. Reg Sci Urban Eco. (1988) 18:549–66. 10.1016/0166-0462(88)90026-9

[41] Hsu C Fan C Mostafavi A. Limitations of gravity models in predicting fine-scale spatial-temporal urban mobility networks. Phys. Soc. (2021) 2109:1–18.

[42] Liang L Chen M Luo X Xian Y. Changes pattern in the population and economic gravity centers since the reform and opening up in China: the widening gaps between the South and North. J Clean Prod. (2021) 310:1–13. 10.15957/j.cnki.jjdl.2022.02.011

[43] Ahrens A Lyons S. Do rising rents lead to longer commutes? A gravity model of commuting flows in Ireland. Urban Studies. (2021) 58:264–79. 10.1177/0042098020910698

[44] Beyer RM Schewe J Lotze-Campen H. Gravity models do not explain, and cannot predict, international migration dynamics. Human Soc Sci Commun. (2022) 9:1–10. 10.1057/s41599-022-01067-x

